# The Approach of Fertility Physicians to Donor Sperm Treatments: A Worldwide Survey

**DOI:** 10.5935/1518-0557.20250017

**Published:** 2025

**Authors:** Ettie Maman, Ariel Weissman, Gon Shoham, Jordana Hyman, Yossi Mizrachi, Avi Tsafrir

**Affiliations:** 1 Faculty of Medical & Health Sciences, Tel Aviv University, Tel Aviv, Israel. IVF Unit, Department of Obstetrics and Gynecology, Sheba Medical Center, Ramat Gan, Israel; 2 Faculty of Medical & Health Sciences, Tel Aviv University, Tel Aviv, Israel. IVF Unit, Department of Obstetrics and Gynecology, Edith Wolfson Medical Center, Holon, Israel; 3 Faculty of Medical & Health Sciences, Tel Aviv University, Tel Aviv, Israel. Department of Plastic Surgery, Tel Aviv Sourasky Medical Center, Tel Aviv, Israel; 4 Faculty of Medicine, Hebrew University of Jerusalem. IVF Unit, Department of Obstetrics and Gynecology, Shaare Zedek Medical Center, Jerusalem, Israel

**Keywords:** Donor sperm, cost, Artificial insemination, Natural cycle, intrauterine insemination, survey

## Abstract

**Objective:**

Many women presenting for treatment with donor sperm have never attempted conception, and are therefore presumed fertile. With no clear guidelines, treatment can be influenced by factors like patient age and costs. We aimed to explore fertility physicians’ attitudes and clinical practices regarding donor sperm treatments through a global survey.

**Methods:**

We conducted an anonymous international web-based survey of fertility clinicians. The survey addressed various aspects of donor sperm treatment in women without previous infertility.

**Results:**

A total of 211 clinicians participated, with 63% working in private settings. The survey presented clinical scenarios for women aged 32, 37, and 40. Most clinicians (80%) recommended artificial insemination with donor sperm in a natural cycle (NC-AID) as the first-line treatment for a 32-year-old woman (52% suggested up to 3 cycles, 28% up to 6 cycles). This approach was favored by 45% for a 37-year-old and 25% for a 40-year-old. Variability was noted regarding the second-line treatment for a 32-year-old after one failed NC-AID varied (equal recommendations for ovarian stimulation with oral agents, gonadotropins, or IVF). For older women, active management was preferred: 24% recommended gonadotropins and 15% IVF for 37-year-olds, and 16% gonadotropins and 55% IVF for 40-year-olds.

**Conclusions:**

This survey highlights the controversy surrounding the optimal approach for women with no history of infertility seeking to conceive with donor sperm. Our findings emphasize the need for further research and the development of comprehensive guidelines in this area.

## INTRODUCTION

The use of donor sperm for single women, same-sex couples, and heterosexual couples is on the rise worldwide (Gerkowicz *et al.*, 2018; HFEA, 2021). While originally intended for use in couples with severe male infertility, the main utility of donor sperm at present is among single women and lesbian couples (Arocho *et al*., 2019; Diego *et al.*, 2022).

For women presumed to be fertile, utilizing donor sperm without further intervention is anticipated to yield favorable outcomes. Theoretically, as infertility is conventionally defined as the absence of pregnancy after a year of unprotected intercourse, a one-year trial of intra-uterine inseminations (IUIs) is expected to serve as an alternative to natural intercourse before proceeding to additional interventions, such as ovarian stimulation or in-vitro fertilization (IVF).

Nevertheless, many women attempt to conceive with donor sperm at an advanced reproductive age, where the probability of a live birth decreases significantly (Steiner & Jukic, 2016). Under such circumstance, some may opt for a medical intervention alongside insemination, such as ovarian stimulation or IVF at an earlier stage (HFEA, 2022).

Another potential factor influencing women’s decisions, as well as clinicians’ recommendations, could be the substantial costs associated with the use of donor sperm. The financial consideration might therefore influence the decision on therapeutic approach, favoring methods that are considered more efficient in achieving a live birth, such as adding ovarian stimulation or IVF.

Surprisingly, clear guidelines on the use of donor sperm for presumably fertile women are lacking. Therefore, the treatment in these cases is left to the decision and experience of the caring physician. This study aimed to explore the views and opinions of clinicians on this matter and to examine their therapeutic approach to common clinical scenarios.

## MATERIALS AND METHODS

We conducted a web-based international survey among fertility physicians, using the IVF-Worldwide platform. IVF-Worldwide.com is non-commercial website that has an advisory board of key opinion leaders in the field of IVF. The platform enables access to a large number of clinicians and IVF clinics all over the world, possessing a wide spectrum of opinions. As a result, it is an excellent tool for conducting large-scale surveys that depict trends, common practices, and unanswered questions.

A 13-item survey entitled ‘‘Donor sperm - what do you recommend?’’ was compiled by the authors, who are experienced fertility specialists, and posted on the IVF-Worldwide Website from January 2022 through March 2022. The survey questions can be accessed at: https://ivf-worldwide.com/survey/donor-sperm-what-do-you-recommend.html. The survey questions focused on the management of patients undergoing treatments with donor sperm in various clinical scenarios.

As the survey does not involve research on human subjects, formal approval from an institutional review board was not required. The survey was accessible as an open-access questionnaire on IVF-worldwide.com, and participants voluntarily responded to the study questions. All data collected for this research remained anonymous.

## RESULTS

In this survey, 211 physicians from 52 countries, actively participating in artificial insemination with donor sperm (AID) treatments, filled out the questionnaire. Respondents were asked to estimate the number of donor sperm cycles they perform annually. The results represent 10550 annual donor sperm cycles. The geographic distribution of participating IVF units, with their corresponding estimated annual number of IVF cycles is presented in Table 1. Detailed responses to all questions given by all respondents can be accessed through the IVF-Worldwide Web site at https://ivf-worldwide.com/survey/donor-sperm-what-do-you-recommend/results-donor-sperm-what-do-you-recommend.html.

**Table 1:** Geographic distribution of survey participants.

Continent	Respondents (%)	AID Cycles/year(%)
USA & Canada	24 (11.5)	1490 (14.1)
South America	37 (17.5)	1190 (11.3)
Australia & New Zealand	6 (2.8)	320 (3)
Asia	52 (24.6)	2485 (23.6)
Europe	81 (38.4)	4835 (45.8)
Africa	11 (5.2)	230 (2.2)
Total	211 (100)	10550 (100)

AID, artificial insemination with donor sperm.

Among the respondents, 63% exclusively practice in private settings, 10% practice in public settings, and 27% engage in both. Regarding patient volumes, 21% of clinics serve fewer than 10 patients utilizing donor sperm annually, 46.9% serve 10-50 patients, 16.5% serve 50-100 patients, and 15.2% serve more than 100 patients utilizing sperm donations annually.

**Figure 1:**
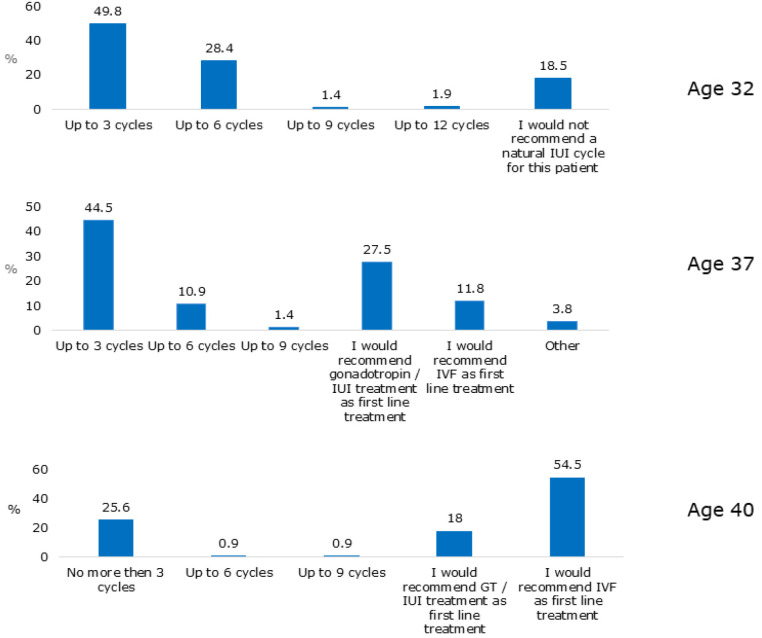
Survey respondents’ recommendations for the number of natural cycle assisted insemination with donor sperm cycles for patients with normal ovarian reserve and no infertility history, across different ages. GT - Gonadotropins, IUI - Intrauterine insemination.

Survey participants were asked to outline their primary suggestion for women with a normal ovarian reserve assessment and no prior infertility issues, aiming to conceive with donor sperm. Regarding natural cycle (NC) AID, the recommendations provided by respondents exhibited a significant correlation with patient’s age. In this scenario, 49.8%, 44.5%, and 25.6% of the respondents recommended up to 3 NC-AID cycles in 32, 37 and 40 year-old patients, respectively. This trend was also maintained regarding the option to perform more than six or nine NC-AID cycles (Figure 1).

While the majority of respondents favored attempting NC-AID for up to 6 cycles in 32-years-old patients, 18.5% indicated they would not advocate NC-AID attempts at all. Sub-analysis revealed that while 4.8% of respondents working exclusively in public practices would not recommend NC-AID in this scenario, 22% respondents working exclusively in private settings would not recommend NC-AID in this scenario.

When presenting the same clinical scenario, but with a 37-year-old patient, the majority of respondents also supported up to 6 cycles of NC-AID. However, almost 40% supported advanced treatments like gonadotropin-stimulated AID or IVF as the first choice. Practice type had a lesser effect on the response. The impact of patient’s age was maintained in 40-year-old patients, where advanced treatment with IVF was recommended as the initial treatment by 54.5% of respondents, again with lesser effect of practice type (63% exclusively working in private practice).

**Figure 2:**
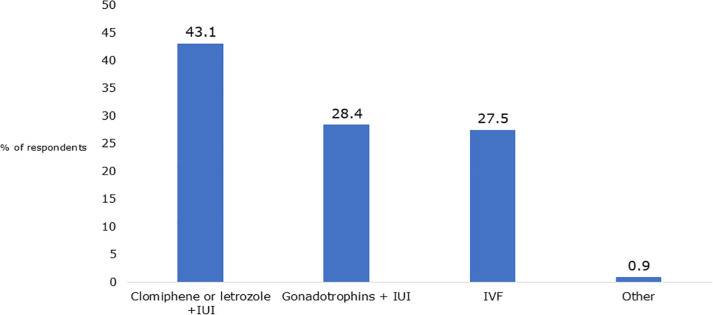
Survey respondents answers to the question “What treatment would you recommend to a 32-year-old patient who did not conceive after natural cycle donor insemination?”.

Subsequently, we examined the approach taken when an initial attempt with spontaneous cycles and IUI did not result in pregnancy. Initially, respondents were asked about their recommendations for 32-year-old patients following unsuccessful NC-AID. There was a notable divergence of opinions, with outcomes nearly evenly distributed across three treatment categories. Specifically, 43% of respondents favored recommending ovarian stimulation using Clomiphene Citrate or Letrozole combined with IUI, 28% suggested administration of gonadotropins, and an additional 28% advocated proceeding with IVF (Figure 2).

**Figure 3:**
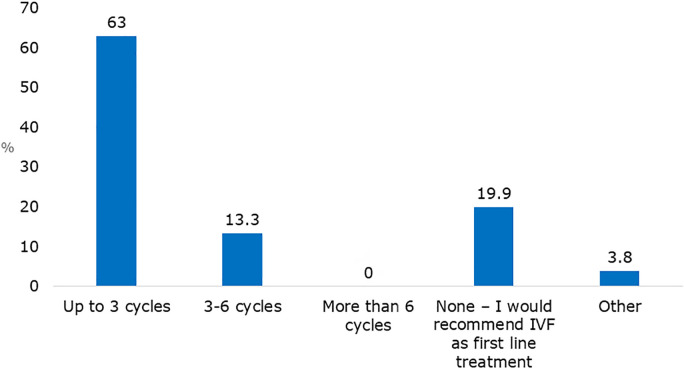
Survey respondents answers to the question “How many gonadotropin-donor-IUI cycle would you recommend to a 37-year-old patient before proceeding to IVF?”.

In response to the question, “How many gonadotropin-IUI cycles would you recommend a 37-year-old patient before opting for IVF?” 63% of respondents would attempt up to 3 cycles, while 13% would consider 3-6 cycles. However, a notable 20% would avoid this option altogether, instead recommending IVF as the primary treatment approach (Figure 3).

The subsequent query aimed to assess respondents’ perspectives on the need for infertility workup in patients who wish to conceive with donor sperm. Participants were asked regarding their recommendation of hysterosalpingography or hydrosonography for a 37-year-old woman intending to conceive with donor sperm, with no history or sonographic indications of tubal pathology. Notably, 75% responded affirmatively.

**Figure 4:**
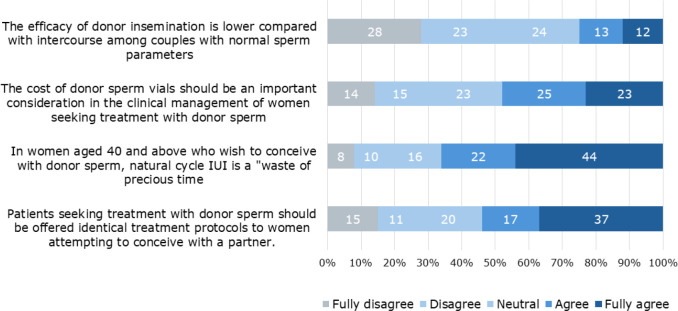
Survey respondents agreement levels with statements regarding treatment with donor sperm.

Finally, respondents were requested to indicate their level of agreement, using a 1-5 scale, with statements related to treatment involving donor sperm. The majority of respondents (67%) expressed a high level of agreement (rating 4 and 5) with the statement, “In women aged 40 and above who wish to conceive with donor sperm, natural cycle IUI is a ‘waste of precious time’.” This agreement is in correlation with the previous question regarding the recommended initial treatment. The three statements, which addressed whether treatment protocols for donor and partner’s sperm should be identical, the consideration of the cost of donor sperm in clinical management, and the efficacy of donor sperm insemination compared to natural intercourse received varied responses (Figure 4).

## DISCUSSION

The current survey highlights ongoing controversies over the optimal approach for women seeking donor sperm treatment, without known infertility. The key issue is whether to treat them as having fertility problems. Many respondents consider these patients as such, with age playing a major role in treatment decisions. Most favor early use of hormonal stimulation or IVF as the patient’s age increases, even without diagnosed fertility issues. However, there was inconsistency in the recommended number of treatment cycles, approach after one failed NC-AID attempt, and the need for infertility diagnostics, reflecting a lack of consensus. While there are established guidelines on the management of various infertility diagnoses, the use of donor sperm for presumably fertile women is seldom addressed. For example, an American Society of Reproductive Medicine document provides detailed recommendations for evaluating potential sperm donors and their recipients but does not address aspects of the clinical use of sperm donors (Practice Committee of the American Society for Reproductive Medicine, 2021). Our literature search yielded only two published guidelines addressing treatment with donor sperm. The British National Institute for Health and Care Excellence 2013 guidelines, updated in 2017, suggests at least six unstimulated AID cycles for women who are ovulating (NICE, 2017). The Practice Committee of the American Society for Reproductive Medicine suggests performing - evaluation and initiating treatment sooner than 12 months for women under 35 years of age and 6 months for women age 35 or older when using cryopreserved donor sperm (Practice Committee of the American Society for Reproductive Medicine, 2020). However, we could not detect more detailed and specific guidelines for further management of women and couples using donor sperm.

In this study, participants were presented with scenarios involving women without known infertility. For single women using donor sperm, AID substitutes natural intercourse. Despite more than 50% of respondents agreeing that “patients seeking treatment with donor sperm should be offered identical protocols to those conceiving with a partner,” many recommended a more active approach. Half advised no more than three natural cycle IUI attempts for women aged 32, while 73% recommended IVF or medicated IUI for women aged 40 on the first trial. Additionally, 75% would refer a 37-year-old for tubal assessment without clinical suspicion. These recommendations differ from those typically given to women starting natural conception with a partner, suggesting a tendency to manage donor sperm patients like those with infertility. Respondents may believe stimulated AID or IVF is more efficient than NC-AID, despite the lack of prospective studies comparing these methods in presumably fertile women. An RCT is needed to compare non-medicated AID, stimulated AID, and IVF in different age groups. At present, we can only rely on conflicting data extracted from retrospective reports describing outcomes of lesbian and single women to assist in counseling and decision making. In such women, pregnancy rates per cycle were approximately 15% per NC-AID cycle in women at their mid-30s (Carroll & Palmer, 2001; Thijssen *et al.*, 2017). Ferrara *et al.* (2002) reported that pregnancy rate per cycle were 14% and 4% in women aged 35-40 and over 40, respectively, using AID in a natural cycle. The use of ovarian stimulation failed to show any significant improvement over spontaneous ovulation in that retrospective study (Ferrara *et al.*, 2002). De Bruker et al. reported on 1654 women who used AID, of whom 70% received stimulation with Clomiphene citrate, and 13% with gonadotropins. Delivery rate in the first cycle in women at age 35-37 was 20%, and the expected cumulative delivery rate after four cycles was 55%. These outcomes were 13% and 37% in women at age 38-39, and 6 and 20% at age 41. The authors concluded that there was no effect of adjustment for ovarian stimulation on the cumulative delivery rates. Carpinello *et al.* (2021) showed a slight advantage for added stimulation in AID cycles. In a large retrospective work that included 6192 cycles, a 4% increase in the CPR rate and only 1% in LBR was observed with the addition of ovarian stimulation compared to natural cycles. However, a significantly increased multiple gestation rate was observed as well. It is important to note that letrozole and clomiphene citrate were used for ovarian stimulation and the authors do not indicate the average number of ovarian follicles during the treatments. Taken together, the tendency of doctors to include ovarian stimulation in AID treatments is clearly not supported by the available scientific data for women with no recognized infertility.

In general, IVF results using donor sperm are comparable to or better than the results obtained using partner sperm (Allen *et al.*, 2023; Catalini *et al.*, 2023). While we could not detect information on outcomes of women who had IVF for the sole reason of using donor sperm according to age, these can be estimated by using calculators such as one provided by the American Society for Assisted Reproductive Technology (SART, 2024). According to this source, expected cumulative delivery rates in the first IVF cycle due to male factor infertility (i.e., assuming no fertility problem of the female patient) cycle at age 35, 37 and 40% are 47%, 37 and 20%, respectively. While these data do not allow us to directly compare IUI to IVF in these women using AID with no infertility, IVF seems to be more effective if one compares them to the reported success rates of both natural and stimulated AID treatments (Carroll & Palmer, 2001; Thijssen *et al.*, 2017). While Evans *et al.* (2024) claimed to achieve satisfactory cumulative pregnancy rate in up to 8 consecutive ovarian stimulation and IUI treatment cycles, a study by Viloria *et al.* (2011) showed higher cumulative pregnancy rates in IVF for single patients using donor sperm compared to donor insemination with ovarian stimulation (38.2% vs 22.6%, respectively). Furthermore, in many cases, patients seeking donor sperm treatments tend to be older (Viloria *et al.*, 2011) and by using IVF, we also provide the option of storing frozen embryos for future biologically identical siblings. Indeed, in the United Kingdom, almost 60% of patients in female same-sex relationships seeking fertility treatment started IVF without any prior AID cycles in 2018 (HFEA, 2022).

Respondents may believe that medical interventions are justified in order to reduce number of cycles needed to achieve pregnancy and therefore reduce the costs of donor sperm used in natural ovulatory inseminations. Among young women, private practice doctors recommended more advanced treatments than those in public systems. This trend did not appear in older women.

Another possible explanation for respondents’ ‘active’ management of sperm donor treatments in this survey is assuming that thawed donor sperm is less efficient than partners’ sperm and therefore women using it should be managed differently. However, only 25% of respondents agreed with the statement that “The efficacy of donor insemination is lower compared with intercourse among couples with normal sperm parameters”. Indeed, donor sperm was reported to be non-inferior to partner’s sperm in IVF success rates (Bortoletto *et al*., 2021) and early pregnancy outcomes (Allen *et al.*, 2021).

Finally, we can speculate that the psychological situation of women attempting to conceive with donor sperm differs from those trying with a partner. Even without hormonal stimulation or IVF, the basic requirements for ovulation monitoring and the insemination procedure itself constitute a form of fertility treatment, carrying a recognized psychological burden. Additionally, clinicians may consider stimulated IUI or IVF to reduce time-to-pregnancy, which is crucial for patients (Roque & Simon, 2020).

Our findings highlight knowledge gaps in the clinical management of donor sperm treatments. For instance, at what point during multiple trials, and at what age, do interventions like ovarian stimulation or IVF become more effective than unstimulated IUI? How do cost calculations vary across health systems? Additionally, what are patients’ preferences? For example, would a 38-year-old opt for 2-3 cycles of unstimulated IUI, or prefer a single IVF attempt, which may be quicker and similarly effective but carries additional costs and risks?

A major strength of this study is its focus on a commonly overlooked yet relevant clinical issue. The inclusion of clinicians from various countries provides a broad, global perspective. However, the study has limitations, particularly the low response rate. The use of closed-ended questions in the online survey limited the depth of responses compared to methods like interviews or focus groups, hindering a deeper exploration of respondents’ choices. Additionally, sampling and non-response biases may affect the survey’s generalizability.

In conclusion, this survey underscores the ongoing challenges in managing women using donor sperm, considering both clinical and cost-related factors. Most respondents approached these cases as they would with infertility patients, rather than focusing solely on the absence of a male partner. Our findings highlight the need for further research and the development of comprehensive guidelines in this area.
